# Longitudinal Brain White Matter Alterations in Minimal Hepatic Encephalopathy before and after Liver Transplantation

**DOI:** 10.1371/journal.pone.0105887

**Published:** 2014-08-28

**Authors:** Wei-Che Lin, Kun-Hsien Chou, Chao-Long Chen, Hsiu-Ling Chen, Cheng-Hsien Lu, Shau-Hsuan Li, Chu-Chung Huang, Ching-Po Lin, Yu-Fan Cheng

**Affiliations:** 1 Department of Diagnostic Radiology, Kaohsiung Chang Gung Memorial Hospital and Chang Gung University College of Medicine, Kaohsiung, Taiwan; 2 Brain Research Center, National Yang-Ming University, Taipei, Taiwan; 3 Department of Surgery, Kaohsiung Chang Gung Memorial Hospital and Chang Gung University College of Medicine, Kaohsiung, Taiwan; 4 Department of Biomedical Imaging and Radiological Sciences, National Yang-Ming University, Taipei, Taiwan; 5 Department of Neurology, Kaohsiung Chang Gung Memorial Hospital and Chang Gung University College of Medicine, Kaohsiung, Taiwan; 6 Department of Internal Medicine, Kaohsiung Chang Gung Memorial Hospital and Chang Gung University College of Medicine, Kaohsiung, Taiwan; University of Queensland, Australia

## Abstract

Cerebral edema is the common pathogenic mechanism for cognitive impairment in minimal hepatic encephalopathy. Whether complete reversibility of brain edema, cognitive deficits, and their associated imaging can be achieved after liver transplantation remains an open question. To characterize white matter integrity before and after liver transplantation in patients with minimal hepatic encephalopathy, multiple diffusivity indices acquired via diffusion tensor imaging was applied. Twenty-eight patients and thirty age- and sex-matched healthy volunteers were included. Multiple diffusivity indices were obtained from diffusion tensor images, including mean diffusivity, fractional anisotropy, axial diffusivity and radial diffusivity. The assessment was repeated 6–12 month after transplantation. Differences in white matter integrity between groups, as well as longitudinal changes, were evaluated using tract-based spatial statistical analysis. Correlation analyses were performed to identify first scan before transplantation and interval changes among the neuropsychiatric tests, clinical laboratory tests, and diffusion tensor imaging indices. After transplantation, decreased water diffusivity without fractional anisotropy change indicating reversible cerebral edema was found in the left anterior cingulate, claustrum, postcentral gyrus, and right corpus callosum. However, a progressive decrease in fractional anisotropy and an increase in radial diffusivity suggesting demyelination were noted in temporal lobe. Improved pre-transplantation albumin levels and interval changes were associated with better recoveries of diffusion tensor imaging indices. Improvements in interval diffusion tensor imaging indices in the right postcentral gyrus were correlated with visuospatial function score correction. In conclusion, longitudinal voxel-wise analysis of multiple diffusion tensor imaging indices demonstrated different white matter changes in minimal hepatic encephalopathy patients. Transplantation improved extracellular cerebral edema and the results of associated cognition tests. However, white matter demyelination may advance in temporal lobe.

## Introduction

Hepatic encephalopathy (HE) is frequently associated with a wide range of neuropsychiatric abnormalities in liver cirrhosis, and has been classified as a continuum from minimal HE (MHE) to different grades of overt HE. [1] It is believed that cerebral edema is the common pathogenic mechanism for cognitive impairment in MHE and overt HE. [2], [3] Although patients with MHE present as essentially normal, such patients perform abnormally on psychometric tests and have an increased risk of motor vehicle accidents. Liver transplantation (LT) can correct liver function, resulting in an improvement symptom of MHE. [4] However, at least during the first 2 years after LT, some cognitive defects seem to persist to some degree. [5] Thus, whether complete reversibility of brain edema, cognitive deficits, and their associated imaging can be achieved remains an open question.

The MHE patients can experience persistent cognitive deficits after LT. [6] In addition, permanent brain injury with volume atrophy has been found in those with previous episode of overt HE. [6] Each of these observations suggests that cirrhosis may cause brain damage that persists after LT. Some prospective imaging studies have shown improvement in HE related brain edema [7], [8] after treatment, with such improvement being primarily due to removal of interstitial type brain edema. [8] However, it is also common for cirrhotic patients to experience acute liver failure or overt HE accompanied by irreversible cytotoxic cerebral edema. [9] The interaction or evolution between two forms of cerebral edema complicates the interpretation of their effects on cognition outcomes after LT, a subject which has not been studied in depth before.

Diffusion tensor imaging (DTI) provides information about different direction of water mobility, as well as information about tissue microstructure and organization, through different quantitative DTI metrics. In quantitative terms, mean diffusivity (MD), an index of averaged diffusivity across three-dimensional space, and fractional anisotropy (FA), an index of the microstructural integrity of the brain’s white matter (WM), have been widely used as quantitative metrics in previous studies. [10] Recently, the directional diffusivities, axial diffusivity (D_ax_ = λ_1_) and radial diffusivity [D_rad_ = (λ_2_+λ_3_)/2], have been used to demonstrate the advantage of obtaining additional information about the potential underlying pathophysiology of WM changes. Alterations in D_ax_ suggest the presence of axonal damage and/or Wallerian degeneration in the primary fiber orientation, [11] while increased D_rad_ implies demyelination or dysmyelination. [12] The widely used DTI indices, FA and MD, are associated with cognitive impairment in MHE patients before LT. [8], [13] However, there are no reports of longitudinal neuro-imaging studies investigating changes in WM in MHE patients before and after LT through the use of multiple diffusion indices. There is a paucity of knowledge regarding the associations between clinical evaluations and the characteristics of WM integrity evolution in MHE patients receiving LT.

To elucidate the underlying WM microstructural changes occurring in MHE patients, we used voxel-wise analysis of multiple diffusivity indices before and after LT in this longitudinal study, and compared the results with data from healthy volunteers. We sought to determine (1) whether MHE patients show deterioration of WM integrity after LT by assessing multiple DTI indices (2) whether changes in WM integrity either affected by baseline or interval changes in liver functions after LT and (3) whether there is any correlation between WM integrity and cognitive performance before and after LT.

## Materials and Methods

### 1. Participants

A consecutive series of patients with liver cirrhosis who were evaluated for LT were recruited to the study. The project was approved by Chang Gung Memorial Hospital’s Institutional Review Committee on Human Research, and all subjects gave written informed consent before participating. All participants were informed that the study was designed to evaluate manifestations of MHE both before and at 6 to 12 months after LT.

The diagnosis of liver cirrhosis was based on a consistent clinical history, radiological studies, and liver biopsy when available. [8] Patient functional status was assessed using the Child-Pugh scoring system. [14] According to Ferenci’s report, [15] MHE was evaluated by the Wechsler Adult Intelligence Scale III (WAIS-III) subtests, including the digit-symbol and block design subtests. [16] A test result was considered abnormal for the digit-symbol and block design subtests if it was 2 standard deviations below the mean score of normal subjects. Thirty age- and sex-matched normal subjects (19 men, 11 women; 52.80±9.77 years) without any medical history of neurological disease served as the control group and were recruited by advertisement within the hospital. The mean digit-symbol score for the normal controls was 57.28±13.52 and the block design score was 40.50±10.86. Therefore, cirrhotic patients with a digit-symbol score of 30 or lower or with a block design score lower than 19 were recognized as having MHE.

Laboratory screening, MRI scans, and neuropsychiatric tests were performed on the same day for all patients. Initially, 70 cirrhotic patients who completed the neuropsychological evaluation and MRI examinations were enrolled in the study. Participants were excluded if they were diagnosed with overt HE or produced normal neuropsychological tests results. Any history of drug abuse, psychiatric or neurological illness, head injury, or poor image quality with severe distortion and metallic artifacts that might compromise diffusion tensor image analysis was also grounds for exclusion.

Finally, 28 adult cirrhotic patients with MHE (24 men, 4 women; 51.14±8.38 years) were included in this study, and 42 patients were excluded (normal neuropsychiatric test results, *n* = 29; overt HE, *n* = 9; poor image quality, *n* = 4).

### 2. Neuropsychological tests

A battery of neuropsychological tests, which focused on attention, executive function, speech and language function, and visuo-construction function, was performed. Different domains of neuropsychological evaluations were measured by subtests from Cognitive Ability Screening Instrument (CASI), [17] WAIS-III [18] and *Wisconsin Card Sorting Test* (WCST-64, Computer Version Scoring Program). [19].

### 3. MRI data acquisition

Images were obtained using a 3.0-T whole body GE MR system (Signa, General Electric Healthcare, Milwaukee, WI, USA) with a standard eight-channel phase-array head coil. Participant head movement inside the coil was minimized immobilization with cushions. Whole brain DTI images were acquired using an axial single-shot spin-echo diffusion-weighted echo-planer imaging sequence, with array spatial sensitivity encoding to reduce susceptibility and eddy-current distortions. The DTI imaging parameters were as follows: repetition time (TR)/echo time (TE) = 15800/77 ms, number of excitations (NEX) = 3, field of view (FOV) = 256 mm^2^, slice thickness = 2.5 mm, matrix dimensions = 128×128, 55 slices without gaps, b-value = 1000 s/mm^2^, 13 non-collinear diffusion directions and a non-diffusion-weighted T2 images (b-value = 0 s/mm^2^). The DTI session design was based on the balancing of diffusion gradients to minimize eddy-current artifacts.

Structural images were acquired using standard T1-weighted three-dimensional fluid-attenuated inversion recovery fast spoiled gradient-recalled echo pulse sequence with the following imaging parameters: TR/TE/TI = 9.5/3.9/450 ms; flip angle = 15 degrees; NEX = 1; field of view = 240*240 mm^2^; slice thickness = 1.3 mm; matrix size = 512*512; voxel size = 0.47*0.47*1.3 mm^3^ and 110 contiguous slices that aligned to the anterior commissure-posterior commissure.

Additional whole brain axial T2-weighted, and fluid-attenuated inversion-recovery fast-spin-echo sequences were applied to define anatomical details. One author blinded to participant status visually checked all MRI scans to confirm that the participants were free from morphological abnormalities. The total MRI scanning time was approximately 23 min for each participant. The mean duration between MRI acquisition at baseline and follow up for the patient group was 8.43 months (range: 6.5 to 12 months).

### 4. DTI data preprocessing and voxel-wise WM microstructure investigations

Image data was analyzed by a researcher with 8 years of experience in MRI research using FSL v5.0.4 (Functional Magnetic Resonance Imaging software obtained from the Brain Software Library; http://fsl.fmrib.ox.ac.uk/fsl/fslwiki/). [20] Each diffusion-weighted image was affine registered to the non-diffusion-weighted image to correct the head motion and eddy-current distortion. To further minimize motion effects in DTI estimation, each applied gradient direction of the diffusion-weighted image was reoriented to the corresponding transformation matrix that described the rotation parameters of subject motion. [21] After skull stripping, diffusion indices were calculated by fitting a tensor model using FMRIB’s Diffusion Toolbox. Longitudinal voxel-wise analysis of WM microstructure was performed using Tract-based Spatial-Statistic (TBSS), [22] which has been described in detail in previous study. [23] First, all subjects’ FA maps were non-linearly warped to the Montreal Neurological Institute (MNI) space FMRIB58_FA template using FMRIB’s nonlinear registration tool. Next, MNI space FA maps were averaged and thinned to generate a mean FA skeleton; we defined the WM skeleton threshold at an FA value greater than 0.2 to further reduce the partial volume effects. Each individual’s MNI space FA map was projected onto the mean threshold skeleton and the resulting data were subsequently fed into the statistical analysis framework. The same registration procedures were applied to MD, D_ax_, and D_rad_. An experienced neuroradiologist visually checked all warped indices to ensure the accuracy of the whole automatic image-preprocessing pipeline.

### 5. Group comparisons of T1 DARTEL VBM

T1 voxel based morphometry (VBM) with the diffeomorphic anatomical registration through exponentiated lie algebra (DARTEL) registration scheme was used for investigating gray matter (GM) volume changes between study groups. [24], [25] Individual T1 scan were analyzed with the VBM8 toolbox (http://dbm.neuro.uni-jena.de) with default settings under Statistical Parametric Mapping (SPM8; Wellcome Institute of Neurology, University College London, UK). The detailed T1 DARTEL VBM pipeline was the same with previous published study from our groups. [26] In briefly, T1 structural scans were bias-corrected, tissue segmented [segmented into GM, WM and cerebrospinal fluid (CSF) compartments], and spatial normalized to MNI space using affine and high dimensional DARTEL registration approach. To conserve the total amount of GM before and after spatial normalization, the nonlinear deformation parameters of the spatial normalization procedure were used to modulate the GM tissue segments and resized voxel sizes to 1.5×1.5×1.5 mm^3^. This non-linear only modulation procedure allow us to make inferences on regional GM volume measurements rather than tissue density (concentration) and further ensure that statistical comparisons are made on relative (controlling for overall brain size) rather than absolute volumes. The resultant MNI space modulated GM segments were smoothed with a Gaussian kernel of 8 mm full-width Gaussian kernel at half-maximum (FWHM) and served as inputs for further voxel-wised group comparisons in GM volume.

The longitudinal T1 DARTEL VBM preprocessing module with default setting in the VBM8 toolbox was used for investigating the longitudinal GM volume changes before and after liver transplantation in patients with cirrhotic. The following preprocessing steps were used in this analysis: (1) the follow-up measurement scan was registered to the baseline measurement scan for each participants; (2) the mean T1 anatomical scan were calculated from the realigned images from previous step of each participant and served as a reference image for subsequent spatial registration; (3) intra-subject bias correction for each time-point realigned T1 anatomical scans were performed with regard to the reference mean image; (4) the bias-corrected mean anatomical scan and realigned scans were segmented into GM, WM and CSF respectively; (5) spatial normalization parameter of DARTEL registration algorithm were estimated using the tissue segments (GM and WM) of the bias-corrected mean anatomical scan; (6) the DARTEL normalization parameters were applied to the GM tissue segments of bias-corrected realigned anatomical scan for each time-point. Finally, the resulting MNI space GM tissue segments were smoothed with 8 mm FWHM Gaussian kernel which was also used in the previous cross-sectional analysis.

### 6. Statistical analysis

All statistical analyses of demographic data were performed using the SPSS (version 12, SPSS Inc., Chicago, IL, USA) with appropriate tests. Age and sex were compared between the study groups by the independent t-test and Pearson’s Chi-square test. Laboratory data were compared between the patient groups before and after LT by the pair t-test. The neuropsychological tests were compared between groups by using independent t-test or the pair t-test with correcting the multiple comparison problem using false discovery rate (FDR). The statistical significant level for neuropsychological tests were set as corrected P_FDR_ <0.05.

#### Group comparisons of diffusion tensor index

For image-based voxel-wise analysis, non-parametric permutation-based statistical analyses were performed to investigate regional WM changes between baseline and follow-up scans of the patient and control group (analysis of covariance model with age and sex as covariates of no interest), and the longitudinal changes before and after LT in the MHE group (paired *t* test). 5000 permutations were used for each possible statistical contrast, and the results were corrected for multiple comparisons across space using a threshold-free cluster enhancement (TFCE) approach with family wised error (FWE) corrected P value <0.05. [27] Mean DTI indices representing each significant cluster were calculated for each participant and further correlated to clinical evaluations. Data was adjusted for age and sex, and the relationships between regional diffusivity indices, cognitive function, and laboratory test results were investigated using partial Pearson correlation analysis. After Bonferroni correction for the number of ROIs, the significance threshold for the two-tailed partial correlation tests was set to a p-value of less than 0.05 (the p-values were adjusted for the number of regions by a factor of 5, similar to an uncorrected p = 0.05/5).

#### Group comparisons of gray matter volume

To ensure that statistical thresholds in T1 DARTEL VBM and DTI TBSS analysis were comparable, the smoothed modulated GM segments were also analyzed using the same statistical design and statistical criteria with a framework of permutation-based non- parametric testing using FSL v5.0.4. The cluster-wised statistic with TFCE clustering approach which used in the DTI-TBSS analysis was used again to determine clusters with significant GM volume changes, and FWE corrected *P value* <0.05 was used to correct for multiple comparisons across space.

## Results

### 1. Clinical characteristics

LT significantly corrected liver function in MHE patients, including prothrombin time (p<0.001), International Normalized Ratio ratio (p<0.001), concentration of albumin (p<0.001), total bilirubin (p = 0.04), and venous ammonia (p<0.001) (Table 1).

**Table 1:** Demographics, clinical characteristics, and cognitive test results of minimal hepatic encephalopathy patients and healthy controls.

	Control	Pre-transplantation	Post-transplantation	p-value	p-value	p-value
Number of subjects	30	28	28	NC vs Pre-LT	NC vs Post-LT	Pre-LT vs Post-LT
Age (years)	52.8±9.8	51.1±8.4	51.1±8.4	0.49	0.49	
Gender (male/female)	19/11	24/4	24/4	0.91	0.91	
Education (years)	11.6±4.1	10.7±3.4	10.7±3.4	0.34	0.34	
Previous hepatic encephalopathy (n)	–	15	15	–	–	
Child-Pugh’s class: A/B/C (n)	–	0/17/9	0/17/9	–	–	
***Biochemical parameters***						
Prothrombin time (seconds)	–	13. 1±3.1	10.4±2.2	–	–	**<0.001**
Albumin (mg/dL)	–	3.4±0.5	4.2±0.5	–	–	**<0.001**
International Normalized Ratio (INR)	–	1.2±0.2	1.1±0.1	–	–	**<0.001**
AST(IU/L)	–	65.8±65.1	64.9±29.5	–	–	0.97
ALT(IU/L)	–	48.5±46.4	47.8±38.4	–	–	0.19
Total Bilirubin (mg/dL)	–	3.0±4.2	1.5±1.7	–	–	**0.04**
Direct Bilirubin (mg/dL)	–	1.6±2.9	0.7±0.2	–	–	0.13
Venous ammonia (µg/dL)	–	125.0±65.5	56.5±48.1	–	–	**<0.001**
**Neuro-psychiatric tests**						
***Attention***						
Mental control (CASI)	9.0±1.3	8.5±2.5	9.2±2.2	0.36	0.68	**0.03**
Attention (CASI)	7.7±0.7	7.4±0.8	7.6±0.7	0.10	0.43	0.16
Orientation (CASI)	17.8±0.5	16.8±2.5	17.8±0.6	0.06	0.89	0.05
***Executive function***						
Digit symbol (WAIS)	57.3±13.5	18.3±5.6	26.7±4.9	**0.004** *	**0.010**	**0.006** *
Abstraction (CASI)	10.7±1.4	8.7±1.8	9.1±2.2	**<0.001** *	**0.001** *	0.30
Number correct (WCST)	39.7±11.6	36.6±13.5	41.0±14.5	0.44	0.72	**0.018**
Total error (WCST)	24.4±11.6	27.4±13.5	23.0±14.5	0.44	0.72	**0.018**
Perseverative response (WCST)	12.0±6.2	15.0±11.7	13.0±13.9	0.24	0.73	0.16
Perseverative error (WCST)	11.4±5.4	12.6±8.9	11.2±10.2	0.53	0.93	0.24
Non-perseverative error (WCST)	14.3±10.0	14.5±12.0	11.8±10.9	0.98	0.34	0.10
Conceptual level responses (WCST)	43.6±22.4	44.2±27.7	40.20±35.2	0.85	0.77	**0.04**
Category (WCST)	2.2±1.4	2.0±1.6	2.3±1.6	0.61	0.68	0.10
***Memory function***						
Long-term memory (CASI)	9.9±0.4	9.6±2.5	9.7±1.3	0.52	0.31	0.88
Short-term memory (CASI)	10.8±1.5	10.1±1.9	10.3±2.5	0.13	0.48	0.69
***Speech and Language function***						
Language(CASI)	10.1±1.5	9.9±0.4	9.7±1.1	0.40	0.26	0.42
Verbal fluency (CASI)	8.5±1.6	8.2±2.1	8.0±2.3	0.56	0.26	0.43
***Visuospatial function***						
Picture completion (WAIS)	15.6±5.4	13.2±5.8	14.7±5.9	0.12	0.54	**0.007** *
Letter number search (WAIS)	65.2±22.0	49.6±21.3	60.7±19.7	**0.01**	0.40	**<0.001** *
Block design (WAIS)	40.5±10.9	29.3±12.0	37.3±11.2	**0.04**	0.36	**0.009** *
Drawing (CASI)	9.6±1.4	9.6±1.0	9.4±2.0	0.98	0.85	0.69
***CASI total score***	94.2±4.6	88.9±11.4	91.1±11.5	**0.02**	0.16	**0.02**

Demographic data, including age and sex, were compared among the study groups using the two-sample Student’s *t* test, Pearson’s chi-squared test, and paired *t* test, where appropriate, and are reported as means ± standard deviation (SD).

Abbreviations: WASI, Wechsler Abbreviated Scale of Intelligence; CASI, Cognitive Ability Screening Instrument.

Statistical threshold was set at *p*<0.05 (Boldface).

*Stand for the appropriate test passed the statistical criteria set at corrected P_false-discovery-rate_ <0.05.

### 2. Neuropsychological performance

#### 2.1 Differences in cognition performance between MHE and controls before LT

The MHE patient group exhibited significantdisturbances in executive function (Digit symbol, p = 0.004; Abstraction, p<0.001). We also found a worse performance of visuospatial function (Letter-number search, p = 0.01; Block design, p = 0.04) and the CASI global performance score (p = 0.02) but was not survived with correcting for multiple comparisons (FDR<0.05). The group also displayed a trend of lower attention, memory, and language function scores.

#### 2.2 Differences in cognition performance between MHE and controls after LT

After LT, the MHE patients presented with poorer executive function in the abstraction test (p = 0.001) compared to healthy subjects. The MHE group also had a trend of lower memory and language function.

#### 2.3 Longitudinal changes of cognition performance in MHE patients

Longitudinal comparison after LT showed significant improvements in executive function (digit-symbol test, p = 0.006), and visuospatial function (picture completion, p = 0.007; letter-number search p<0.001; block design, p = 0.009).

### 3. Comparisons of multiple diffusivity indices by DTI-TBSS

#### 3.1 Regional WM diffusivity index changes between MHE patients and the control group

An exploratory comparison between Pre-LT and control groups, and between Post-LT and controls showed no significant differences in WM integrity between groups.

#### 3.2 Longitudinal regional WM diffusivity index changes in MHE patients

**Figure 1:**
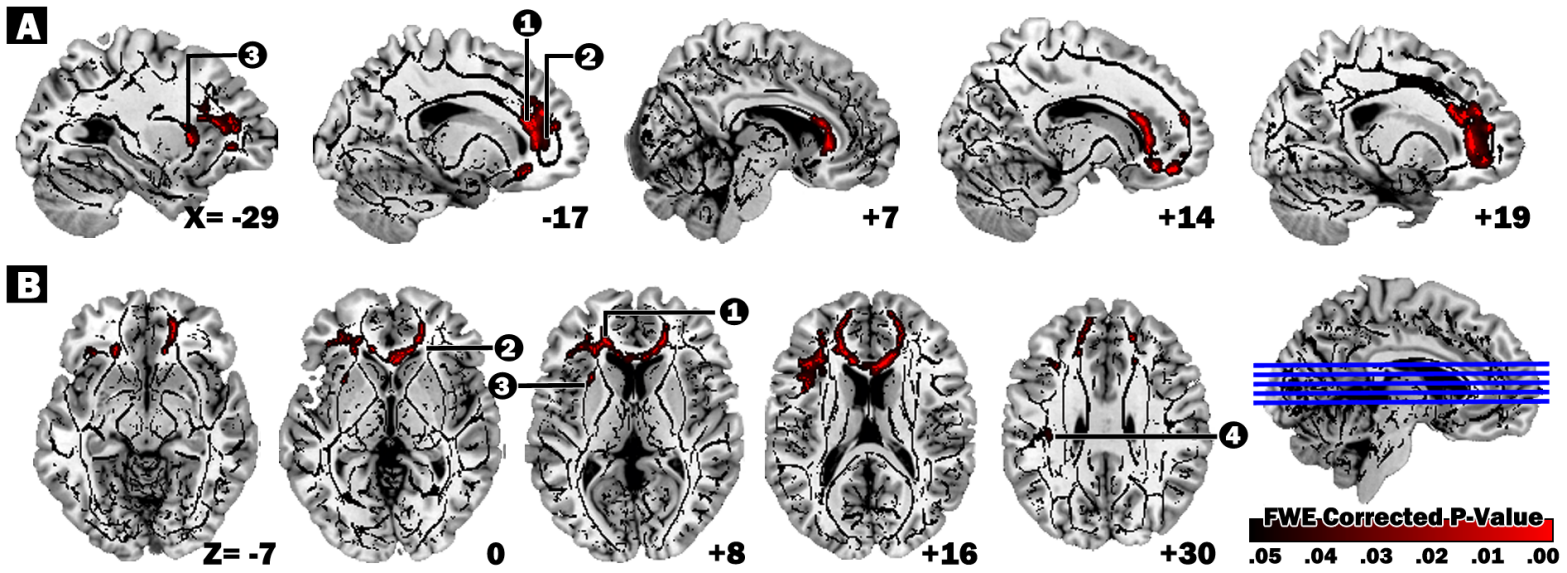
Regions showing significant reductions in axial diffusivity in follow-up scan compared with baseline scan. Regions with decreased axial diffusivity after LT (threshold-free cluster enhancement corrected for multiple comparisons at P_FWE_ <.05). Number 1, Left anterior cingulate; number 2, Right anterior cingulate; number 3, Left claustrum; number 4, Left Postcentral Gyrus; Red maps indicate the degree of the corrected p-value. Anatomical changes are superimposed on the mean WM skeleton and on the T1 template located in MNI space in (A) sagittal and (B) axial views. We used the tbss_fill script to aid visualization. The numbers in the lower right corner of each image represent the MNI (A) x- and (B) z-coordinates respectively. Abbreviations: FWE, family-wise error; MNI, Montreal Neurological Institute.

An exploratory group-wise comparison of the MHE cohort before and after transplantation revealed that post-LT, patients exhibited decreased D_ax_ in several clusters, including WM underlying the left anterior cingulate, right anterior cingulate, left claustrum, and left postcentral gyrus (FWE-corrected p<0.05). Further regional of interest analysis revealed that MD values also decrease in the right anterior cingulate, left claustrum, and left postcentral gyrus (Table 2; Figure 1).

**Table 2:** Regions of longitudinal changes in white matter microstructure between baseline and follow-up scan in MHE patients.

Decreased D_ax_ in follow up vs. baseline scan											
MNI atlas coordinates			Voxels size	White matter tract	Corresponding cortical area	D_ax_ mean (SD)		Z_max_	Other DTI indices (follow up–baseline)		
X	Y	Z				Baseline	Follow up		FA	MD	D_rad_
−18	36	10	1716	Left Limbic Lobe, Anterior Cingulate	Anterior Cingulate, BA32	1.27 (0.08)	1.24 (0.08)	4.32	0.00	−25	−18
19	38	4	1050	Right Frontal Lobe, Corpus Callosum	Anterior Cingulate, BA32	1.18 (0.09)	1.12 (0.07)	4.32	0.00	−27*	−15
−28	13	5	63	Left Sub-lober, Extra-Nuclear	Claustrum	1.26 (0.06)	1.20 (0.06)	5.55	0.00	−35*	−23
−36	−23	27	35	Left Sub-lober, Extra-Nuclear	Postcentral Gyrus, BA2	1.47 (0.05)	1.42 (0.05)	4.81	0.00	−27*	−17

Mean D_ax_ values were directly obtained from clusters showing significant changes between baseline and follow-up scan. Z_max_ values represent TFCE FWE-corrected clusters (p<.05). Multiple brain atlases implemented in FSL were used to define anatomical regions of significant clusters after statistical comparisons. Diffusivity values corresponding to each cluster with significant longitudinal changes are represented as differences (baseline – follow up) in MD and D_rad_ (mm^2^/s) multiplied by a factor of 10^6^. Boldfaced diffusivity values with “*” represent significant differences (*P*<.05, Bonferroni-adjusted) between baseline and follow-up scans.

Abbreviations: FA, fractional anisotropy; MNI, Montreal Neurological Institute; SD, standard deviation; DTI, diffusion-tensor imaging; D_ax_, axial diffusivity; MD, mean diffusivity; D_rad_, radial diffusivity; BA, Brodmann area; TFCE, threshold-free cluster enhancement; FWE, family-wise error.

**Figure 2:**
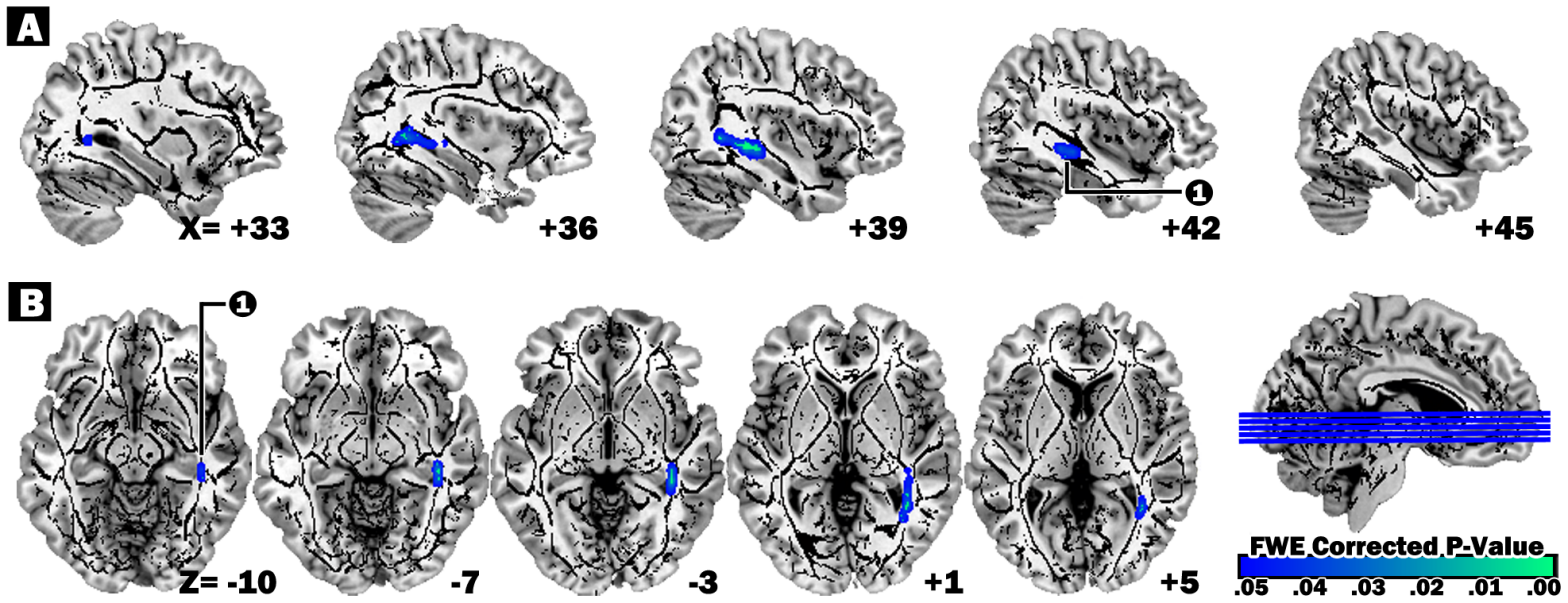
Regions showing significant FA reduction in follow-up scan compared with baseline scan. Regions with decreased FA following LT (threshold-free cluster enhancement corrected for multiple comparisons at P_FWE_ <.05). Number 1, Right parahippocampal gyrus; blue scale color maps indicate the degree of corrected p-value. Anatomical changes are superimposed on the mean WM skeleton and on the T1 template located in MNI space in (A) sagittal and (B) axial views. The tbss_fill script was used to aid visualization. The numbers in the lower right corner of each image represent the MNI (A) x- and (B) z-coordinates respectively. Abbreviations: FWE, family-wise error; MNI, Montreal Neurological Institute.

After LT, the cluster in right temporal lobe had lower FA associated with higher D_rad_ and a trend-level increase in MD among subjects with MHE vs. controls (Table 3; Figure 2).

**Table 3:** Regions of longitudinal changes in white matter microstructure between baseline and follow-up scan in MHE patients.

Decreased FA in follow up vs. baseline scan											
MNI atlas coordinates			Voxels size	White matter tract	Corresponding cortical area	FA mean (SD)		Z_max_	Other DTI indices (follow up–baseline)		
X	Y	Z				Baseline	Follow up		D_ax_	MD	D_rad_
42	−33	−10	178	Right Temporal Lobe, Sub-Gyral	Parahippocampal Gyrus, BA36	0.61 (0.04)	0.59 (0.04)	5.79	−1	21	31*

Mean FA values were directly obtained from clusters showing significant changes between baseline and follow-up scan. Z_max_ values represent TFCE FWE-corrected clusters (p<.05). Multiple brain atlases implemented in FSL were used to define anatomical regions of significant clusters after statistical comparisons. Diffusivity values corresponding to each cluster with significant longitudinal changes are represented as differences (baseline – follow up) in MD, D_ax_ and D_rad_ (mm^2^/s) multiplied by a factor of 10^6^. Boldfaced diffusivity values with “*” represent significant differences (*P*<.05, Bonferroni-adjusted) between baseline and follow-up scans.

Abbreviations: FA, fractional anisotropy; MNI, Montreal Neurological Institute; SD, standard deviation; DTI, diffusion-tensor imaging; D_ax_, axial diffusivity; MD, mean diffusivity; D_rad_, radial diffusivity; BA, Brodmann area; TFCE, threshold-free cluster enhancement; FWE, family-wise error.

### 4. Correlations between clinical laboratory tests and extracted DTI indices in MHE patients

**Figure 3:**
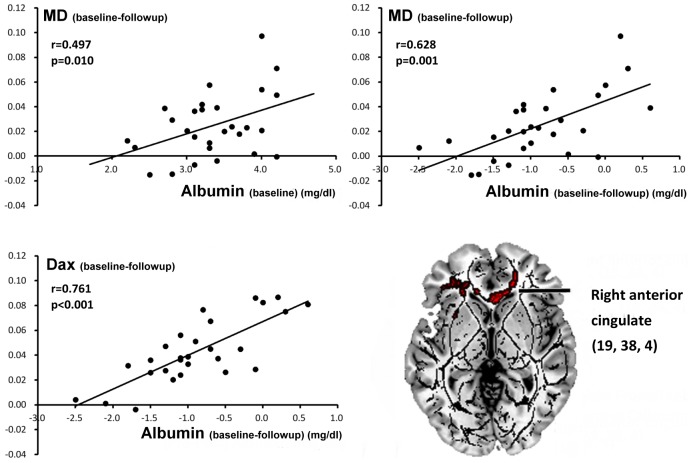
Correlation between albumin level and DTI indices before and after LT. Pearson correlation between interval albumin changes and diffusivity values [D_ax_ and MD, (mm^2^/s)×10^6^] of clusters derived from the comparison of MHE before and after LT. Abbreviations: D_ax_, axial diffusivity; MD, mean diffusivity.

Before LT, higher albumin_(baseline)_ levels were positively associated with better MD_ (baseline-followup)_ recovery in right anterior cingulate (r = 0.50, p = 0.01), indicating better correction of brain edema after transplantation. After LT, we also found that improvement of albumin_(baseline-followup)_ levels were significantly positively associated with recovery of D_rad (baseline-followup)_ (r = 0.76, p<0.001) and MD_(baseline-followup)_ (r = 0.63, p = 0.001) values in right anterior cingulate (Figure 3).

### 5. Correlations between neuropsychological tests and extracted DTI indices in MHE patients

Improvement in *visuospatial function*_(baseline-followup)_ (letter-number search scores) was correlated with interval correction of MD_(baseline-followup)_ values (r = −0.54, p = 0.004) in the right postcentral gyrus, after correction of the multiple comparisons.

### 6. Comparison of T1 DARTEL VBM between cirrhotics patients before and after LT, and healthy subjects

The VBM analysis comparing cirrhotic patients before and after LT, and control subjects included in the study were showed in table S1 and figure S1. Before LT, there was a significant smaller gray matter volume in cerebellum and putamen, and larger gray matter volume in thalamus in cirrhotic patients compared to healthy subjects. After LT, post-LT groups showed smaller gray matter volume only in putamen compared to healthy subjects. Comparison between pre- and post-LT group showed recovery of the gray matter volume occurred in postcentral gyrus, cerebellar tonsil and inferior frontal gyrus. Our results were partially supported previous findings. [28], [29].

## Discussion

We used TBSS with multiple diffusivity indices to investigate specific information about the pathogenesis of observed WM changes in MHE patients during pre-LT and post-LT periods. We demonstrated that MHE patients might undergo different types of WM changes before and after LT. Improved WM indices in MHE patients who subsequently experienced cognitive recovery arose primarily from the edematous effect; however after LT, progressive demyelination occurred in temporal WM, a finding which has not been previously reported. The recovery from WM edema, particularly in the right anterior cingulate, is predictable from Pre-LT albumin levels, and albumin_(baseline-followup)_. Our results showed that most of the tissue microstructure changes in patients with liver cirrhosis are reversible.

Evaluation of multiple DTI indices facilitated our investigation into the histology of WM and the possible etiologies of disease evolution in vivo. After LT, the regional decreases in MD in the bilateral anterior cingulate, left claustrum, and left postcentral gyrus are driven by decreased D_ax_ levels, and not by decreased D_rad_. Significant decreases in D_ax_, and an MD index with unchanged FA and D_rad_ values after LT suggested decreased tissue water diffusivity without changes to fiber integrity or cell damage. Chavarria et al performed a biexponential analysis of DTI, and reported that MD increased from a “fast diffusion pool” that returned to normal after LT, and concluded that cerebral edema is majorly extracellular. [30] However, intra-astrocytic accumulation of osmotic materials had promoted astrocyte swelling as a common pathogenetic endpoint of chronic liver cirrhosis. Our results do not fully explain the movements of cerebral water, but may indicate the coexistence of extracellular and intracellular edema in MHE. Thus, histologically tolerable myelin and axonal loss without reactive astrocytosis may allow the reversibility of DTI indices after LT. [31].

Six months after LT, patients exhibited significant improvements in all cognitive domains, except for memory, speech, and language functions. The DTI indices improved mainly for the frontal and parietal lobes and for the limbic system, which are important for most executive, coordination, and visuospatial functions. Not all cognitive recovery is directly associated with the observed improvements in WM DTI indices, and changes in DTI indices might result from other contributing factors, such as reductions in gray matter edema or gray matter atrophy in present and other study. [28], [29] However, the significant correlation between decreased MD, especially in the postcentral gyrus, and improvement in visuospatial function after LT provides support for our conclusion that chronic HE affects the superior parietal and posterior frontal convexities most significantly, [31] which are responsible for the identification and visuospatial location of a stimulus. [32].

The concentration of albumin in plasma is usually low in patients with cirrhosis and HE, and greater change in albumin levels results in better DTI indices. The administration of human albumin in patients with hepatorenal syndrome and spontaneous bacterial peritonitis has a major impact on the prognosis of these complications. [33] The mechanism of action may involve the maintenance of oncotic pressure and scavenging of toxic substances present in blood, which reduces the risk of intracellular edema and neurological injury. [34] In addition, lower albumin levels may occur in cases of malnutrition, which has been linked to decreased DTI indices in liver cirrhosis. [35] Our findings suggest that microstructural changes that result from cerebral edema might arise from decreases in albumin levels, and may reverse during clinical recovery.

Another important finding was a progressive decline in FA in the right temporal lobe, even after LT. Decreased FA has been reported for patients with low grade HE before receiving LT, [13] and decreased WM density in the temporal lobe had also been observed in patients with cirrhosis. [28] The extension and size of the affected areas is greater, a greater degree of liver failure with the antecedent of overt HE is more common in patients with alcoholic cirrhosis. [28] These effects can continue even after LT; the parahippocampus and the hippocampus are extremely sensitive to hypoxia. [36] Episodes of HE usually lead to swelling of astrocytes, cytotoxic edema, and intracranial hypertension, and such occurrences increase the stress of hypoxia. In fulminate HE, significant decreases in MD and FA values failed to recover in a follow-up study, [9] suggesting that damage from hypoxia with cytotoxic edema might increase vulnerability to irreversible WM damage in the temporal lobe. In addition, immunosuppressive agent used to prevent graft-versus-host disease has also been associated with several side-effects, including hypertension and neurotoxicity. Although the mechanism of their neurotoxicity are not well known, cerebral vasculopathy [37] and hypertensive encephalopathy [38] had been associated to brain injury and some of the toxicity from particular immunosuppressive agent can mainly manifest as white matter brain lesions. Longitudinal, multiple sections evaluation might help to clarify the effects from immunosuppressive agents to brain microstructure in the future. In the present study, we interpreted decreased FA and increased D_rad_ in the temporal lobe, without changes in D_ax_, as demyelination and gliosis, consistent with histological findings. [31] The WM in MHE patients might present with mixed effects, [30] from longstandingvasogenic edema secondary to cirrhosis, episodic acute cytotoxic edema, secondary to hyperammonemic decompensation and/or neurotoxicity effect from immunosuppressive agent.

In the present study, the changes in WM integrity were as subtle as the cognition deficits in MHE patients when compared with healthy subjects. The healthy condition in patients with minimal HE is quite good showing limited cognitive decline before operation. This is somewhat reasonable that we could not find any difference between the patient group and healthy controls with the conservative statistical threshold. We reported more strictly statistical criteria which may increase specificity, but also reduces the sensitivity necessary for detecting subtle temporal changes in WM. In addition, cerebrovascular small-vessel degeneration in the temporal lobe and other WM could not be totally excluded in MHE [39] because an interval-imaging study was not performed on the normal groups, however, changes in the WM of healthy participants over a short duration can be limited.

## Conclusions

Our longitudinal voxel-wise multiple DTI indices study revealed various WM changes, including improvement of the extracellular cerebral edema and of the demyelination of vulnerable WM that occur in MHE patients accepting liver transplantation. DTI may be useful for investigating the pathogenesis of MHE for adequate interpretation of cognitive impairment and for assessing the efficacy of therapeutic measures focused on correcting this disorder.

## Supporting Information

Figure S1 **Regions showing significant gray matter volume changes between cirrhotic patients before/after liver transplantation, and healthy subjects.** Different color maps show the cluster-level statistics with the FWE-corrected p values of the corresponding group comparison using TFCE approach. (a), (b) and (c) shows regions of significant gray matter volume changes in cirrhotic patients before/after liver transplantation compared with the NC group. (d) and (e) shows regions of significant longitudinal gray matter volume changes between cirrhotic patients before and after liver transplantation. All of the above results are displayed at the MNI T1 template. Abbreviation: FWE: family-wise error; L: left; MNI: Montreal Neurological Institute; NC: normal control; R: right; TFCE: threshold-free cluster enhancement.
(TIF)

Table S1 **Anatomical regions with significant gray matter volume changes between cirrhotic patients before/after liver transplantation, and healthy subjects using voxel based morphometry approach.**
(DOC)
