# Supplementary figures and images for: Longitudinal Brain White Matter Alterations in Minimal Hepatic Encephalopathy before and after Liver Transplantation
Source: PLoS One. 2014 Aug 28;9(8):e105887. doi: 10.1371/journal.pone.0105887 (PMC4148329; doi:10.1371/journal.pone.0105887)

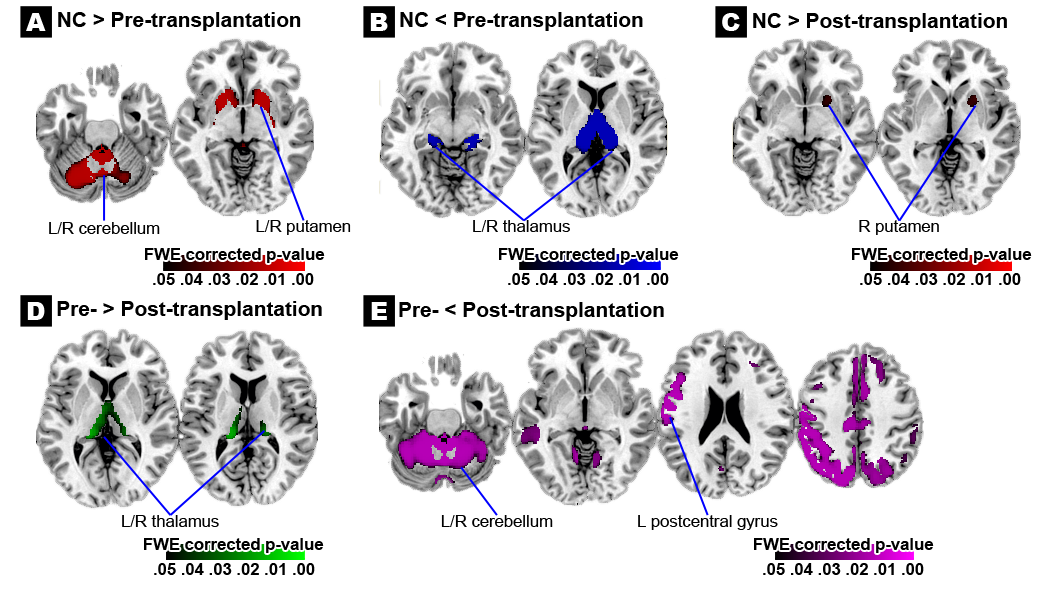

Supplement: Figure S1 — Regions showing significant gray matter volume changes between cirrhotic patients before/after liver transplantation, and healthy subjects. Different color maps show the cluster-level statistics with the FWE-corrected p values of the corresponding group comparison using TFCE approach. (a), (b) and (c) shows regions of significant gray matter volume changes in cirrhotic patients before/after liver transplantation compared with the NC group. (d) and (e) shows regions of significant longitudinal gray matter volume changes between cirrhotic patients before and after liver transplantation. All of the above results are displayed at the MNI T1 template. Abbreviation: FWE: family-wise error; L: left; MNI: Montreal Neurological Institute; NC: normal control; R: right; TFCE: threshold-free cluster enhancement. (TIF) [file pone.0105887.s001.tif]
