# Supplementary material for: Longitudinal Brain White Matter Alterations in Minimal Hepatic Encephalopathy before and after Liver Transplantation
Source: PLoS One. 2014 Aug 28;9(8):e105887. doi: 10.1371/journal.pone.0105887 (PMC4148329; doi:10.1371/journal.pone.0105887)
Supplement: Table S1 — Anatomical regions with significant gray matter volume changes between cirrhotic patients before/after liver transplantation, and healthy subjects using voxel based morphometry approach. (DOC) [file pone.0105887.s002.doc]

**Supplementary Table 1**

Anatomical regions with significant gray matter volume changes between cirrhotic patients before / after liver transplantation, and healthy subjects using voxel based morphometry approach

| **Montreal Neurological Institute atlas coordinates** | | | **Voxels size** | **Anatomic Region** | **T_max_** |
| --- | --- | --- | --- | --- | --- |
| **X** | **Y** | **Z** |
| **Normal controls > Pre-transplantation** | | | | |  |
| -23 | -62 | -32 | 6222 | Left Cerebellum | 5.69 |
| -18 | 5 | -9 | 1638 | Left Putamen | 4.25 |
| 20 | 6 | -9 | 1598 | Right Putamen | 6.17 |
| -20 | -50 | -54 | 113 | Left Cerebellum | 5.32 |
| **Normal controls < Pre- transplantation** | | | | |  |
| 23 | -26 | -8 | 7256 | Right thalamus | 4.82 |
| **Normal controls > Post- transplantation** | | | | |  |
| 26 | 12 | -3 | 207 | Right Putamen | 4.94 |
| **Pre- > Post- transplantation** | | | | |  |
| -17 | -32 | 8 | 1702 | Left Thalamus | 6.35 |
| **Pre- < Post- transplantation** | | | | |  |
| -56 | -9 | 18 | 25442 | Left Postcentral Gyrus | 4.92 |
| -30 | -39 | -41 | 17557 | Left Cerebellum | 4.48 |
|  | | | | | |
